# Recent advances in ternary Z-scheme photocatalysis on graphitic carbon nitride based photocatalysts

**DOI:** 10.3389/fchem.2024.1359895

**Published:** 2024-04-03

**Authors:** Dantong Zhou, Dongxiang Li, Zhi Chen

**Affiliations:** ^1^ College of Electronic and Information Engineering, Anshun University, Anshun, China; ^2^ College of Materials and Chemistry, China Jiliang University, Hangzhou, China

**Keywords:** G-C_3_N_4_, ternary composite photocatalysts, all-solid-state ternary Z-scheme, direct ternary Z-scheme, application

## Abstract

Due to its excellent photocatalytic performance over the last few years, graphitic-like carbon nitride (g-C_3_N_4_) has garnered considerable notice as a photocatalyst. Nevertheless, several limitations, including small surface area, the rates at which photo-generated electrons and holes recombine are swift, and the inefficient separation and transport of photoexcited carriers continue to impede its solar energy utilization. To overcome those limitations in single-component g-C_3_N_4_, constructing a heterogeneous photocatalytic system has emerged as an effective way. Among the various studies involving the incorporation of hetero composite materials to design heterojunctions, among the most promising approaches is to assemble a Z-scheme photocatalytic configuration. The Z-scheme configuration is essential because it facilitates efficient photocarrier separation and exhibits superior redox ability in separated electrons and holes. Moreover, ternary composites have demonstrated enhanced photocatalytic activities and reinforced photostability. Ternary Z-scheme heterostructures constructed with g-C_3_N_4_ possess all the above-mentioned merits and provide a pioneering strategy for implementing photocatalytic systems for environmental and energy sustainability. A summary of the latest technological advancements toward design and fabrication in ternary all-solid-state Z-scheme (ASSZ) and direct Z-scheme (DZ) photocatalysts built on g-C_3_N_4_ is presented in this review. Furthermore, the review also discusses the application of ternary Z-scheme photocatalytic architecture established on g-C_3_N_4_.

## 1 Introduction

The evolution of socioeconomic and industrial landscapes has given rise to substantial hurdles in the realms of energy assets and ecological sustainability. Perpetual use of fossil resources has triggered the greenhouse effect and resource scarcity (Acar and Dincer, 2014; Li et al., 2020). Simultaneously, the presence of dyes, antibiotics, heavy metal-based chemicals, and other organic contaminants in sewage poses significant hazards to both public health and ecological wellbeing (Zhang et al., 2019; Yang et al., 2021a). At present, semiconductor photocatalysis technology can efficiently utilize sustainable and renewable solar energy as a sustainable driving force to alleviate these pressing resource and environmental issues (Hisatomi et al., 2014; Moroz et al., 2018; Zhang et al., 2019). Since Fujishima and Honda proposed the photoelectrocatalytic hydrogen production through TiO_2_ in 1972, photocatalytic technology developed based on semiconductor materials has commenced to flourish (Fujishima and Honda, 1972). In recent years, photocatalysts have found extensive applications across diverse fields, such as photocatalytic degradation to remove pollutants (Yang et al., 2021b), carbon dioxide reduction (Park et al., 2021), hydrogen production (Hisatomi et al., 2014), and oxygen production (Wang Z. et al., 2019).

The discovery of exceptionally successful photocatalysts holds significant importance for advancing and applying photocatalytic technology. Numerous semiconductors including TiO_2_ (Ma et al., 2014), ZnO (Wolcott et al., 2009), CdS (Tada et al., 2006), Fe_2_O_3_ (Moniz et al., 2015), and BiVO_4_ (Hong et al., 2011) have garnered extensive research attention as photocatalysts. In 2009, Wang et al. (2009b) took the pioneering step of employing g-C_3_N_4_ functioning as a photocatalyst for the generation of hydrogen through photocatalysis. In the investigation undertaken by Wang et al. (2009b), g-C_3_N_4_ demonstrated excellent performance in hydrogen generation devoid of noble metals. Consequently, g-C_3_N_4_ has garnered increasing interest as a prospective visible light reactive photocatalyst. With its excellent chemical stability, strong responsiveness to visible light, cost-effective synthesis process, easy modification, and non-toxicity, g-C_3_N_4_ emerges as an outstanding photocatalyst (Wang et al., 2012; Ong et al., 2016). However, practical utilization of g-C_3_N_4_ as a photocatalytic substance faces significant constraints due to drawbacks such as a low specific surface area, inadequate dissociation of photoinduced charge carriers, and the capacity to harness light in the visual spectrum below 460 nm, leading to reduced solar light utilization efficiency (Jia et al., 2023). Therefore, it is imperative to identify an appropriate approach to improve its performance in photocatalysis. The Z-scheme photocatalysis demonstrates excellent efficiency in separating photoinduced electrons and holes, and these photoexcited charge carriers exhibit outstanding redox capabilities (Kausar et al., 2022). Combining different photocatalysts to establish a Z-scheme photocatalysis setup has become one of the most attractive solutions over the last few years (Kumar et al., 2020). Compared to the composite of two distinct semiconductor materials to form Z-scheme heterojunction photocatalysts, ternary composite semiconductor materials exhibit better visible light responsiveness, higher charge transfer efficiency, and better stability (Xie and Zhang, 2018; Beyhaqi et al., 2020).

**FIGURE 1:**
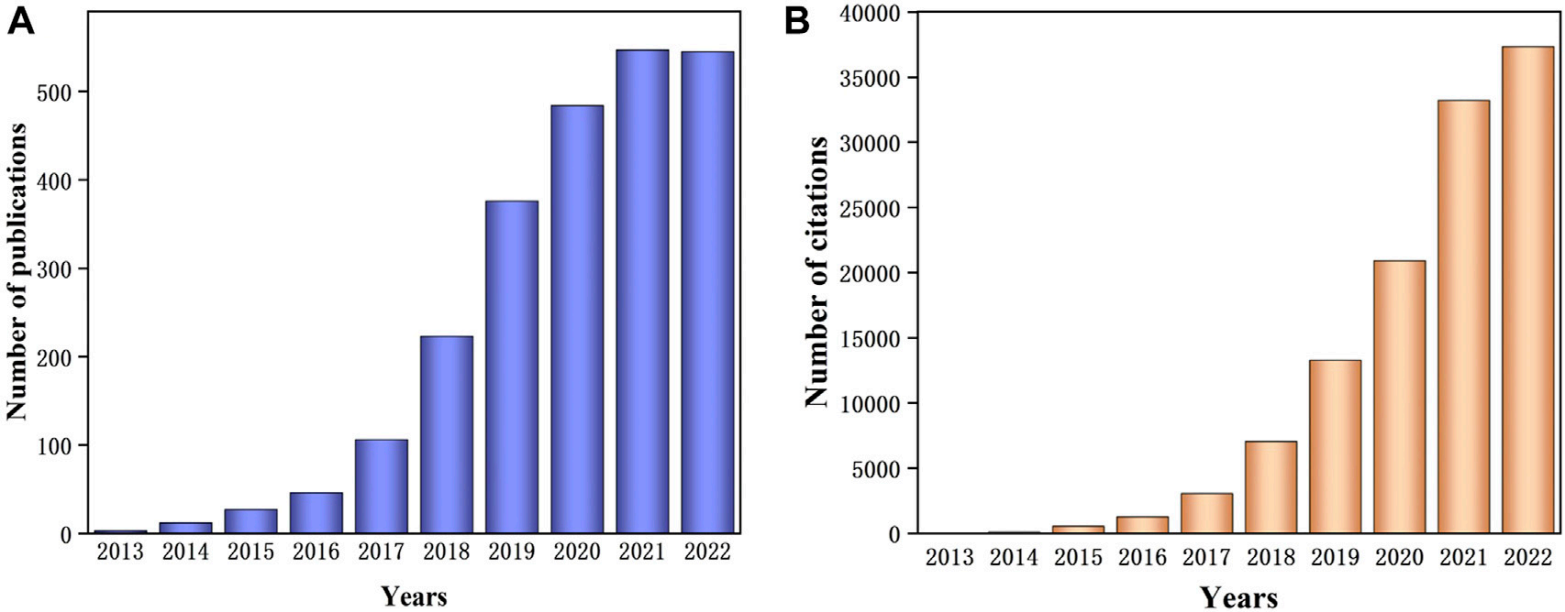
The annual number of **(A)** publications and **(B)** total number of citations including keywords “g-C_3_N_4_” and “Z-scheme” on indexed “Web of Science” between 2013 and December 2022.

According to the information retrieved from Web of Science by Clarivate Analytics (Figure 1), the research concentrated on Z-scheme photocatalytic design utilizing g-C_3_N_4_ incrementally increasing year by year. The emergence of Z-scheme photocatalytic systems formed by g-C_3_N_4_ is clearly becoming an optimal solution to tackle both environmental and energy challenges Research on modification strategies of Z-scheme photocatalytic design established on g-C_3_N_4_ is also abundant, and one highly promising direction in this research involves constructing photocatalysts composed of three semiconductor materials with g-C_3_N_4_ as one of the components. However, there are relatively few reviews specifically centered on the subject involving ternary composite photocatalysis based on g-C_3_N_4_ in a Z-scheme configuration. This review seeks to present an overview of the most recent advancements in ternary heterojunction photocatalysts based on g-C_3_N_4_ in a Z-scheme design, placing a focus on the preparation and principles of ternary ASSZ and DZ photocatalytic systems. Additionally, a concise discussion of the practical applications of ternary Z-scheme photocatalysis on g-C_3_N_4_-based photocatalysts is incorporated.

### 1.1 Characteristics and development of g-C_3_N_4_

Polymeric graphitic carbon nitride (briefly referred to as g-C_3_N_4_), which reveals a structure resembling graphite and is interconnected by both triazine (C_3_N_3_) and tris-triazine/heptazine (C_6_N_7_) rings, is one of the seven types of C_3_N_4_ (Zheng Y. et al., 2015; Jia et al., 2023). Among the various carbon-nitride allomorphs, the first discovered one is g-C_3_N_4_, which is the most stable under common atmospheric parameters However, it has not been utilized as a photocatalyst for a very long time. Since 2009, g-C_3_N_4_ has begun to be favored as a highly promising photocatalyst and its research then experienced explosive growth (Wang et al., 2009b; Cao et al., 2015).

The non-toxic and physicochemical stable polymer semiconductor g-C_3_N_4_ can be produced *via* a facile thermal polymerization method by using the low-cost nitrogen-rich and oxygen-free component precursors such as thiourea (Yu et al., 2017), dicyandiamide (Ji et al., 2013), cyanamide (Li et al., 2013), urea (Martin et al., 2014), and melamine (Yan et al., 2009; Mo et al., 2015). Simultaneously, g-C_3_N_4_ as a graphite-like layered material can be exfoliated to two-dimensional layered material. In recent years, there has also been plenty of research put the spotlight on transforming bulk g-C3N4 into nanosheets through exfoliation, particularly through liquid phase exfoliation (Yang et al., 2013), thermal exfoliation (Xu et al., 2014), ultrasonic exfoliation (Zhao et al., 2014b), chemical exfoliation (Xu et al., 2013), and ultrasonic exfoliation following thermal etching (Zhao et al., 2014a) methods. Furthermore, it is worth noting that controlling the dimension and nanostructure design were also commonly employed research approaches for g-C_3_N_4_. For the reason that the malleable framework structure of g-C_3_N_4_, nanorods (Zeng et al., 2018), nanosheets (Yang et al., 2013), nanospheres (Zheng et al., 2015a), and porous (Wu et al., 2020) g-C_3_N_4_ were satisfactorily obtained. The electronic energy bandgap of g-C_3_N_4_ is 2.7 eV, allowing it to utilize solar energy at wavelengths shorter than 460 nm (Zheng Y. et al., 2015). This makes it an attractive option for a photocatalyst responsive to visible light. At the same time, the attractive energy levels of the conduction band (CB, −1.1 eV) and valence band (VB, +1.6 eV) in g-C_3_N_4_ are appropriate for various photocatalytic reactions (Akhundi et al., 2019), such as hydrogen evolution (Kang et al., 2015), CO_2_ reduction (Pengfei Xia et al., 2017), disinfection (Ma et al., 2016), and pollutant degradation (Jo and Selvam, 2017).

However, several fundamental disadvantages impede the practical application of g-C_3_N_4_. First of all, although g-C_3_N_4_ theoretically possesses a layered two-dimensional arrangement bound by van der Waals forces, its actual specific surface area is quite low. This results in an inability to provide numerous active sites, mitigating the rapid rate of recombination for photoexcited carriers (Niu et al., 2012). Secondly, the effectiveness of photogenerated electron (e^−^) and hole (h^+^) is quite low. Finally, when exposed to visible light, pristine g-C_3_N_4_ can only harvest light with wavelengths below 460 nm, leading to a relatively inefficient utilization of solar (Prasad et al., 2019). As a consequence, attempts to overcome the disadvantages of g-C_3_N_4_ and enhance its performance in photocatalysis through various methods have never ceased in recent years.

### 1.2 Principle of different types of Z-scheme photocatalytic configuration

**FIGURE 2:**
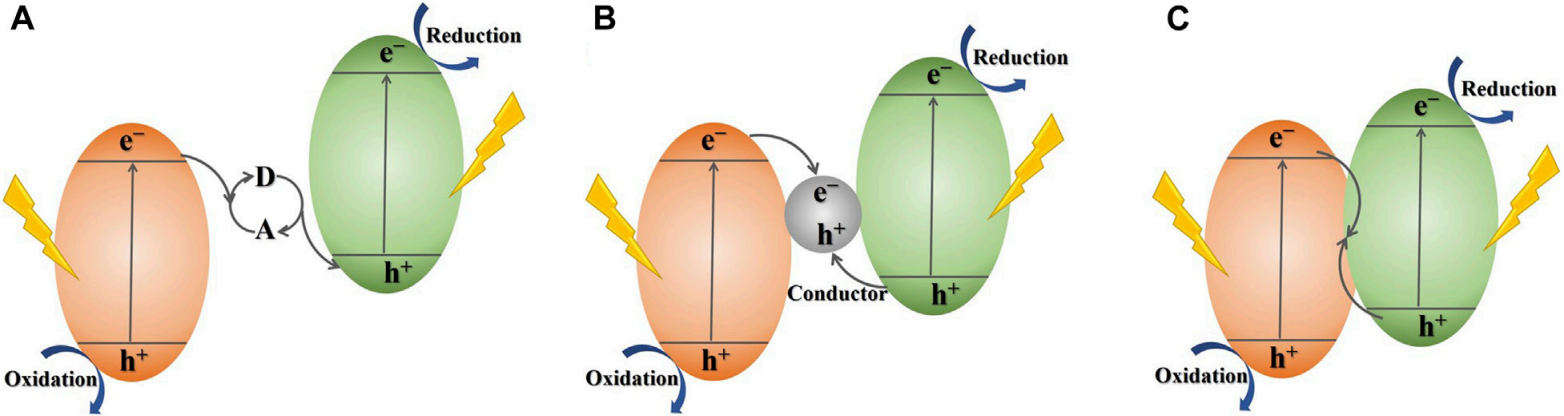
Schematic illustration of different types of Z-scheme photocatalytic systems: **(A)** traditional Z-scheme photocatalytic system, **(B)** all-solid-state Z-scheme photocatalytic system, **(C)** direct Z-scheme photocatalytic system.

In the pursuit of advanced photocatalytic systems that effectively harness solar energy and efficiently separate photogenerated electron-hole pairs, various endeavors have been undertaken, heterojunction construction stands out as a promising and attention-grabbing approach. It is noteworthy that among diverse heterojunctions, Z-scheme photocatalytic system, which simulates the photosynthetic mechanisms in nature, has received the greatest attention in recent years (Zhou et al., 2014). Basically, Z-scheme photosynthesis is designed to mimic the process of photosynthesis that occurs in green plants in nature. As shown in Figure 2A, the photocatalysts with semiconductor heterojunctions in the traditional Z-scheme photocatalytic design will produce photoinduced electrons and holes under a light-excited state, with charge carriers remaining in the CB and VB, respectively. Then, an electron acceptor/donor (A/D) serves as an intermediary for electron transfer. Facilitating the transfer of photoinduced electrons is achieved by utilizing the redox mediators of the A/D pair. This enables the electrons to shuttle from the CB of one photocatalyst to the VB of another (Zhou et al., 2014). Therefore, the retained photo-generated e^−^ and h^+^ in each of the two semiconductor photocatalysts engage in distinct reduction and oxidation processes individually. During this process, the photo-generated e^−^ and h^+^ experience improved charge separation and exhibit enhanced redox potentials when involved in redox reactions, thereby enhancing the photocatalytic performance effectively. However, this traditional Z-scheme system that mimics the natural photosynthesis is always a liquid-phase system due to the A/D pairs are often in a liquid-phase environment, such as IO^3−^/I^−^, Fe^3+^/Fe^2+^ (Ge and Li, 2017). Therefore, this type of Z-scheme photocatalytic configuration is usually liquid-phase. This significantly hinders the utilization of photocatalysts, such as in the photocatalytic decomposition of pollutants, where pollutants can retard the redox reaction of the A/D and affect the photocatalytic performance (Zhou et al., 2014). Consequently, the ASSZ and DZ photocatalytic mechanism design have emerged.

Compared to traditional Z-scheme mechanism with a liquid phase, the absence of a liquid phase in ASSZ and DZ systems offers several advantages in terms of preparation and application and has garnered increased attention and research in the current period. The research on Z-scheme photocatalytic systems has entered a completely new stage since Tada et al. (2006) propounded an ASSZ photocatalytic configuration with Au as an electron shuttle between TiO_2_ and CdS in 2006. As depicted in Figure 2B, the ASSZ mechanism utilizes solid-state conductors as electronic medium, forming the ohmic junction with minimal contact resistance among the pair of semiconductors, and the path of charge carriers transport is comparable to that of a traditional z-scheme setup (Zhou et al., 2014; Deng et al., 2018). In the advancement of ASSZ photocatalytic mechanism, there has been extensive research on using noble metals (Lu et al., 2017), graphene (Xian et al., 2014), and carbon dots (Asadzadeh-Khaneghah et al., 2021) as solid-state electron conduction mediators. The ASSZ mechanism has effectively addressed the stability issues and has broadened the range of applications in photocatalysis.

As the investigation into Z-scheme photocatalytic configuration advances, another new system, the DZ photocatalytic design, has been suggested. The DZ photocatalytic architecture was originally presented by Yu et al. (2013) in 2013. The operation of DZ was shown in Figure 2C, the photoinduced e^−^ transfer directly from one semiconductor to the another through a tightly surface contact of two semiconductors without the need for an electron medium (Low et al., 2017). The DZ photocatalysis setup retains the benefits of elevated efficiency in charge carrier separation and the optimized oxidation-reduction capacity of photogenerated electron-hole pairs, similar to traditional and ASSZ photocatalytic configurations. And it is worth mentioning that the challenges posed by the shielding effect induced by the carrier transport mediators and photo-corrosion will be effectively resolved in a photocatalytic system with a DZ (Xu Q. et al., 2018).

### 1.3 Ternary composite photocatalysts

Composite semiconductor photocatalysts typically possess excellent properties, including a tunable bandgap, increased capture of visible light, and the ability to induce defects to hinder electron-hole pair recombination generated during the assimilation of light (Dahl et al., 2014). Due to its exceptional layered two-dimensional arrangement, g-C_3_N_4_ is beneficial for hybridization with other components and the construction of composite photocatalysts, such as surface coupling and doping with other semiconductors (Zhao et al., 2015). In recent years, ternary composites with g-C_3_N_4_ have been a very encouraging method to broaden the applicability of g-C_3_N_4_. Furthermore, owing to the ability of ternary composite photocatalysts established on g-C_3_N_4_ form double heterojunction or heterojunction-metal structure, they can optimal performance enhancement of g-C_3_N_4_ in photocatalysis more effectively comparison with g-C_3_N_4_-based binary composites (Zhang et al., 2021b). In order to achieve superior performance in terms of visible light reactivity, charge carrier separation, and interfacial charge transfer when compared to binary composite photocatalysts, the g-C3N4 ternary hybrid photocatalysts were designed (Mao et al., 2018; Beyhaqi et al., 2020). In the survey by Xie and Zhang (2018) on ternary Z-scheme photocatalyst based on Ag_3_PO_4_, it was also found that the ternary system provided enhanced electron transfer channels, accordingly efficiently mitigating the photocorrosion of the photocatalyst. In a similarly constructed ternary nanocomposite Ag_2_CrO_4_/g-C_3_N_4_/graphene oxide by Xu D. et al. (2018), it was proposed that the ternary composite system effectively addressed the issue of sluggish electrons and holes transfer and effectively involved photogenerated carriers in the redox reactions, providing more efficient redox reaction sites and making a significant contribution to charge separation. In view of the numerous novel discoveries made in recent years in the field of ternary composite Z-scheme photocatalysts based on g-C_3_N_4_, we believe it is necessary to conduct a review of this subject to accelerate further advancements in this promising research field.

## 2 All-solid-state ternary Z-scheme photocatalysts

### 2.1 Noble metal as electron shuttle

In the draft and construction of an ASSZ photocatalysis setup, the electron shuttle with good electrical conductivity and electron mobility is a considerable situation that requires careful thought. Traditionally, noble metals like Ag, Au, and Pt have served as carrier transport agents. Simultaneously, the loading of noble metals induces the surface plasmon resonance (SPR) effect, efficiently enhancing the visible light region absorption phenomenon by the photocatalysts (Si et al., 2020; Madhusudan et al., 2021). Furthermore, precious metals on the surface of semiconductor nanostructures form Schottky junctions, optimizing the photocatalytic capabilities (Jiménez-Calvo et al., 2020; Yu et al., 2021). The latest advancements g-C_3_N_4_-based all-solid-state ternary Z-scheme photocatalysis, employing valuable metals as electron shuttles, are outlined in Table 1.

**TABLE 1:** Recent progress in g-C_3_N_4_-based ASS ternary Z-scheme photocatalysis with different electron mediators.

PS I (N)	PS II	Electron shuttle	Light source	Application	Activity	Ref
g-C_3_N_4_	MoS_2_	Ag	300 W Xe lamp (λ > 420 nm)	degradation of RhB	DE = 100% (60 min)	**Lu et al. (2017)**
				H_2_ production	104 μmol h^−1^ g^−1^	
g-C_3_N_4_	BiVO_4_	Ag	300 W Xe lamp (λ > 350 nm)	Degradation of TC	DE = 90.76% (60 min)	**Chen et al. (2017a)**
			300 W Xe lamp (λ > 420 nm)		DE = 82.75% (60 min)	
g-C_3_N_4_	NaTaO_3_	Ag	300 W Xe lamp (λ < 420 nm)	Degradation of TC	DE = 95.47% (60 min)	**Tang et al. (2018)**
			300 W Xe lamp (λ > 420 nm)		DE = 91.48% (60 min)	
g-C_3_N_4_	Bi_3_TaO_7_	Ag	300 W Xe lamp	Degradation of SMZ	DE = 98% (25 min)	**Ren et al. (2019)**
g-C_3_N_4_	Ag_3_PO_4_	Ag	300 W Xe lamp (λ > 420 nm)	Removing of NO	74% (90 min)	Li et al. (2021a)
g-C_3_N_4_	BiVO_4_	Ag	300 W Xe lamp (λ > 420 nm)	Degradation of CIP	DE = 92.6% (120 min)	**Deng et al. (2018)**
g-C_3_N_4_	LaFeO_3_	Ag	300 W Xe lamp (λ > 420 nm)	Degradation of MB	DE = 98.97% (90 min) DE = 92.93% (120 min)	**Zhang et al. (2021a)**
				Degradation of TC		
g-C_3_N_4_	AgVO_3_	Ag	300 W Xe lamp (λ > 400 nm)	Degradation of RhB	DE = 100% (12 min)	**Liu et al. (2019)**
				*E. coli* inactivation	3.05 log (100 min)	
g-C_3_N_4_	AgCl	Ag	300 W Xe lamp (λ > 420 nm)	Degradation of Rh B	DE = 100% (60 min)	**Bao and Chen (2016)**
				Degradation of MO	DE = 99% (90 min)	
g-C_3_N_4_	Ag_2_CrO_4_	Ag	500 W Xe lamp	Degradation of MO	DE = 78% (30 min)	**Yu et al. (2021)**
g-C_3_N_4_	TiO_2_	Ag	500 W Xe lamp	Reduction of U (VI)	99% (30 min)	**Liu et al. (2022)**
g-C_3_N_4_	Zn0.5Cd0.5S	Au	300 W Xe lamp (λ > 420 nm)	Reduction of CO_2_ for CH_3_OH evolution	1.31 μmol h^−1^ g^−1^	**Madhusudan et al. (2021)**
g-C_3_N_4_	CdS	Au	300 W Xe lamp (λ > 455 nm)	H_2_ production reduction of CO_2_	277 μmol h^−1^ (4 h)	**Zheng et al. (2015b)**
			300 W Xe lamp (λ > 420 nm)		85%	
g-C_3_N_4_	ZnIn_2_S_4_	Au	300 W Xe lamp	Removal of NO	59.7%	**Zhang et al. (2020a)**
				CO production	242.3 μmol h^−1^ g^−1^	
g-C_3_N_4_	AgCl	Au	200 W Xe lamp (λ > 420 nm)	Degradation of Rh B	DE = 93.1% (25 min)	**Zhang et al. (2021b)**
g-C_3_N_4_	TiO_2_(P25)	Au	150 W Hg Lamp	H_2_ production	419 μmol h^−1^ g^−1^	**Jiménez-Calvo et al. (2020)**
g-C_3_N_4_	Cu_2_ZnSnS_4_	Pt	400 W Xe lamp (λ > 420 nm)	Reduction of CO_2_ for CO/CH_4_ evolution	17.351/7.961 μmol h^−1^ g^−1^	**Raza et al. (2020)**
g-C_3_N_4_	AgVO_3_	Pt	300 W Xe lamp (λ > 420 nm)	H_2_ production	10,444 μmol h^−1^ g^−1^	**Qureshi et al. (2023)**
g-C_3_N_4_	BiVO_4_	Pt	300 W Xe lamp (λ > 420 nm)	Degradation of MB	DE = 100% (70 min)	**Si et al. (2020)**
				Degradation of BPA	DE = 92.7% (130 min)	
				H_2_ production	72 μmol h^−1^ g^−1^	
g-C_3_N_4_	WO_3_	C	500 W Xe lamp (λ > 420 nm)	Degradation of TC	DE = 75% (60 min)	**Zhao et al. (2021)**

DE, degradation efficiency; E, efficiency; Rh B, Rhodamine B; TC, tetracycline; SMZ, sulfamethoxazole; NO, nitric oxides; CIP, ciprofloxacin; MB, methylene blue; MO, methyl orange; BPA, Bisphenol A; 2,4-DCP, 2,4-dichlorophenol; TC-HCl, Tetracycline Hydrochloride; CR, congo red; GO, graphene oxide; RGO, reduced graphene oxide; AFB_1_, Aflatoxins B_1_.

**FIGURE 3:**
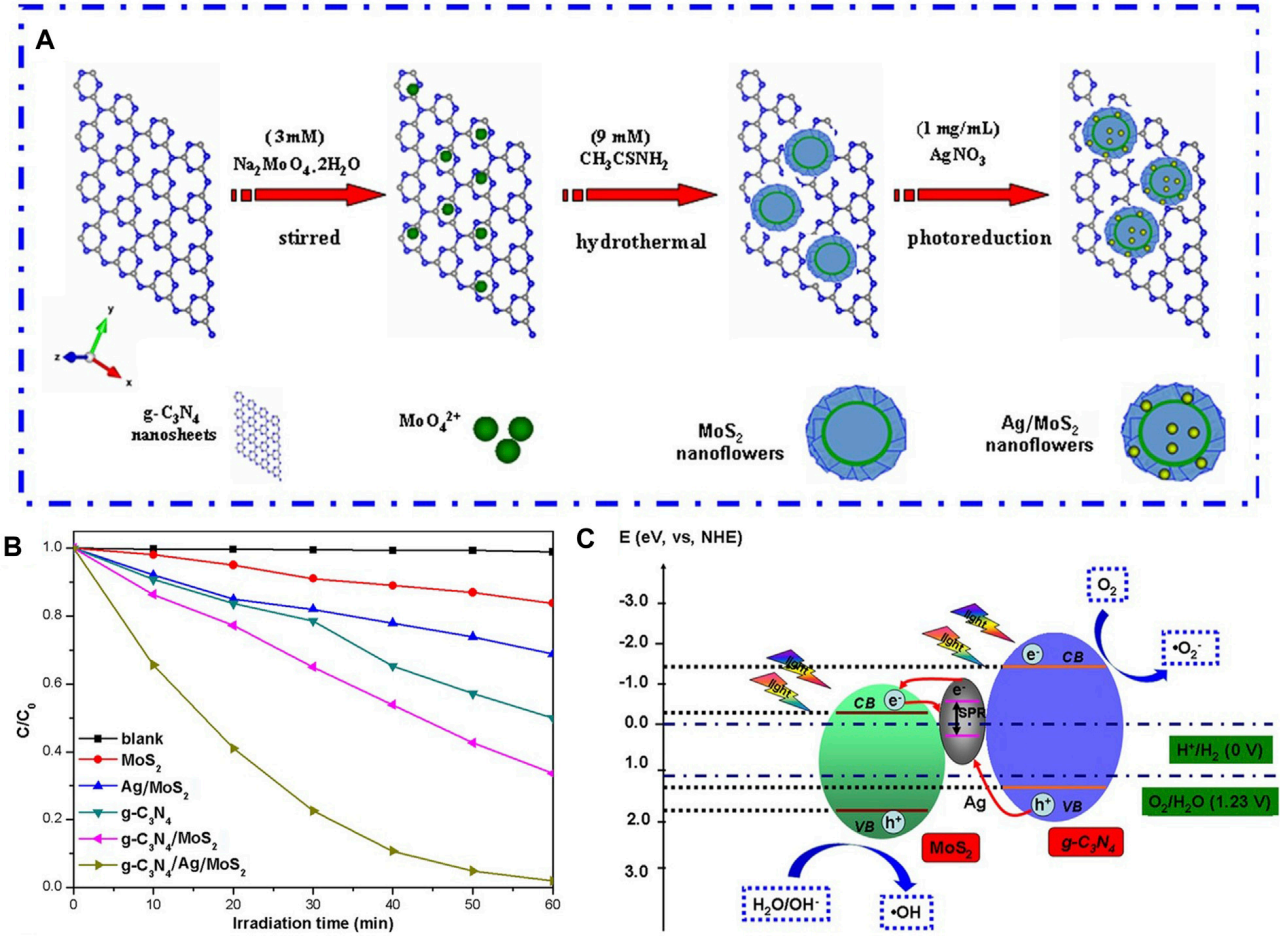
**(A)** Schematic illustration of g-C3N4 and Ag comodified MoS2 microspheres synthesized. **(B)** Visible-light-induced photocatalytic degradation of RhB for different samples. **(C)** Postulate Diagram for the Separation and Transfer of Photogenerated Charges in the g-C3N4/Ag/MoS2 Nanocomposites. Reproduced with permission from Lu et al. (2017), Copyright 2017 American Chemical Society.

With the intention of overcoming the hurdles of quick recombination kinetics of light-induced electron-hole pairs and a limited surface-to-mass ratio of pure g-C_3_N_4_, Lu et al. (2017) proposed a z-scheme ternary photocatalysts g-C_3_N_4_/Ag/MoS_2_ with significantly upgraded visible-light-induced photoactivity. As shown in Figure 3A, the silver (Ag) nanoparticles were decorated on g-C_3_N_4_/MoS_2_ flowerlike microstructure through a photodeposition method, and Ag infiltrated the mesoporous structure within the microspheres composed of g-C_3_N_4_/MoS_2_, positioning itself between g-C_3_N_4_ and MoS_2_ components. The Rhodamine B photocatalytic decolorization and H_2_ generation induced by visible light on g-C_3_N_4_/Ag/MoS_2_ composite were 3.56 and 2.08 fold boost, respectively, as opposed to those on g-C_3_N_4_/MoS_2_ (Figure 3B). This notable enhancement in photocatalytic efficiency under visible illumination is largely credited to the collaborative effects arising from the presence of Ag, g-C_3_N_4_, and MoS_2_, operating within a Z-scheme setup as illumination in Figure 3C. In this system, Ag performs the function of a location facilitating charge transport, where photoinduced e^−^ transfer to metallic Ag from the CB of MoS_2_ and subsequently passes through Ag to amalgamate with h^+^ situated at VB of g-C_3_N_4_. At the same time, the absorption and utilization of visible light by the sample are also enhanced owing to the impact of Surface Plasmon Resonance (SPR) caused by the attendance of Ag. The same g-C_3_N_4_-based ternary structure has also been prepared by other researchers in systems such as g-C_3_N_4_/Ag/NaTaO_3_ (Tang et al., 2018), g-C_3_N_4_/Ag/BiVO_4_ (Chen F. et al., 2017; Deng et al., 2018), gC_3_N_4_/Ag/Bi_3_TaO_7_ (Ren et al., 2019), g-C_3_N_4_/Ag/Ag_3_PO_4_ (Li G. et al., 2021), g-C_3_N_4_/Ag/LaFeO_3_ (Zhang et al., 2021a), g-C_3_N_4_/Ag/Ag_2_CrO_4_(Yu et al., 2021), g-C_3_N_4_/Ag/AgVO_3_ (Liu et al., 2019), g-C_3_N_4_/Ag/AgCl (Bao and Chen, 2016), and g-C_3_N_4_/Ag/TiO_2_ (Liu et al., 2022). In these systems, the placement of Ag is dispersed at the interface boundary of g-C_3_N_4_ and another semiconductor, functions as a link for electronic conveyance to expedite charge transfer. In these ternary Z-scheme photocatalytic configurations based on g-C_3_N_4_, the photocatalytic efficiency when illuminated by visible light has been significantly enhanced as opposed to pristine g-C_3_N_4_ and binary systems.

Analogous to Ag serving as an electron shuttle, Pt is also a commonly employed noble metal as an electron mediator. For example, Raza et al. (2020) prepared a g-C_3_N_4_/Pt/Cu_2_ZnSnS_4_ ternary photocatalyst, which can function as a Z-scheme photocatalyst architecture responsive to visible spectrum for the photochemical reduction of CO_2_ into CO and CH_4_. The CO and CH_4_ yield rates are 3.31 and 5.56 times greater than the values observed for unmodified g-C_3_N_4_. In this composite ternary photocatalyst based on g-C_3_N_4_, the existence of Pt and Cu_2_ZnSnS_4_ results in synergistic effects, including localized surface plasmon resonance, electron sink function, a higher area on the surface, and electron migration in a Z-scheme, all of which collectively enhance the photocatalytic performance.

Additionally, gold (Au) can also act in the capacity of an electron shuttle in a Z-scheme photocatalysis setup. Zhang G. et al. (2020) prepared a Z-scheme photocatalyst, g-C_3_N_4_/Au/ZnIn_2_S_4_, which exhibited the highest efficiency in NO removal and a superior CO production rate when illuminated by visible light. Similarly, Madhusudan et al. (2021) also constructed an ASSZ photocatalyst, g-C_3_N_4_/Zn_0.5_Cd_0.5_S/Au, decorated with Au. This photocatalyst exhibits a 2.9-fold higher photocatalytic CO_2_ reduction efficiency compared to Zn_0.5_Cd_0.5_S/g-C_3_N_4_. Worth mentioning is that at this g-C_3_N_4_/Zn_0.5_Cd_0.5_S/Au photocatalysts, the chemical bonding among these three components is a cornerstone for enhancing the photocatalytic capability of carbon dioxide reduction.

In those semiconductor-metal-semiconductor heterostructures mentioned above, the presence of precious metals plays the role of an e^−^ transfer bridge in Z-scheme photocatalysis, facilitating effective transmission of photoinduced charges, boosting the e^−^ and h^+^ separation, improving photon absorption in the visible light spectrum, and preserving superior oxidation-reduction capabilities of the composite. Consequently, the effectiveness in photocatalysis of Z-scheme photocatalytic design possesses the ability to be significantly enhanced.

### 2.2 Carbon as electron shuttle

Although the precious metal can be worked as an electronic medium in ASSZ photocatalytic configuration, its high price is a limitation that requires consideration. Carbon nanoparticles, as a nontoxic, inexpensive and environmentally friendly material, possess excellent conductivity that is beneficial for charge transfer. They have attracted considerable interest as key constituents in the preparation of ternary Z-scheme photocatalysis incorporating g-C_3_N_4_ as shown in Table 1. Wu and Wang (2021) demonstrated that a carbon film loaded on the exterior of WS_2_ and g-C_3_N_4_ composite exhibits superior photocatalytic effectiveness in comparison to g-C_3_N_4_ and binary g-C_3_N_4_/WS_2_ compound. The photodegradation rate of 2,4-dichlorophenol in the visible spectrum in ternary C@WS_2_/g-C_3_N_4_ composites is about 3.15 and 3.06-fold higher than the values observed for primitive g-C_3_N_4_ and binary WS_2_/g-C_3_N_4_, respectively. In this system, carbon particles measuring 30–50 nm in size, and having an amorphous structure, are coated onto WS_2_, serving as the e^−^ shuttle in Z-scheme photocatalysis. Intriguingly, lacking carbon loading, a type I heterojunction structure forms in binary WS_2_/g-C_3_N_4_, wherein the redox capability of e^−^ and h^+^ is diminished. However, upon the introduction of carbon loading, a Z-scheme structure is established, characterized by electron-hole pairs exhibiting enhanced redox capabilities and superior photocatalytic performance. The incorporation of carbon films between WS_2_ and g-C_3_N_4_ alters the pathway for charge carrier transfer, where carbon acting as an electron transfer shuttle. The CB and VB potentials of g-C_3_N_4_ and WS_2_ were determined by Mott-Schottky experiments, and the photocatalytic mechanism of the photocatalysts was proposed by combining the results of various characterization analyses and photocatalytic performance tests, such as PL, UV-vis DRS, and EPR. Photoinduced e^−^ within the CB of g-C_3_N_4_ no longer directly transport to the CB of WS_2_; instead, photogenerated e^−^ in the CB of WS_2_ is shifted *via* carbon to the VB of g-C_3_N_4_, which recombines there with h^+^. Consequently, ternary C@WS_2_/g-C_3_N_4_ composites that follow the Z-scheme transport mechanism, attain efficient charge differentiation and demonstrate robust redox capabilities. As a result, photocatalytic efficiency under visible-light is significantly enhanced. Notably, not only can the incorporation of carbon onto the semiconductor surface serve as an e^−^ shuttle to facilitate Z-scheme transport, but as demonstrated in the investigation conducted by Zhao et al. (2021), doping carbon into g-C_3_N_4_ can also use carbon as an e^−^ shuttle to establish Z-scheme photocatalysis, thereby enhancing photocatalytic performance. In another reaction involving Cu^2+^ reduction to Cu^+^ in a CuO/CDs/g-C_3_N_4_ ternary photocatalyst employed in a Fenton-like cycle, carbon dots (CDs), serving as dual pathways for charge transfer between CuO/g-C_3_N_4_ heterojunction interfaces, effectively facilitate charge transfer along such Z-scheme system, yielding a synergistic enhancement in photocatalytic performance (Wu et al., 2023).

### 2.3 Graphene oxide as electron shuttle

In the progression of another metal-free ASSZ photocatalyst, graphene oxide (GO), a category of graphene known for its excellent electron conduction efficiency and remarkable two-dimensional carbon sheet structure, can be a superior choice as an electronic shuttle and listed in Table 1 (Xie and Zhang, 2018). In the research of photocatalytic breakdown of contaminants, the existence of graphene oxide not exclusively effectively boosts charge transfer but furthermore provides an extensive reaction region for the adherence and decomposition of target pollutants (Du et al., 2021). In the ASSZ photocatalyst g-C_3_N_4_/GO/AgBr, GO serves in the capacity of a conduit for charge transference connecting two semiconductor materials (Miao et al., 2017). In this ternary photocatalyst, the photocatalytic decolorization of Rhodamine B (Rh B) is 7.9 and 2.2 greater than the value exhibited in g-C_3_N_4_ and binary g-C_3_N_4_/AgBr, respectively. In another g-C_3_N_4_/MnO_2_/GO Z-scheme heterojunction photocatalyst, GO promotes electron transfer and prevents charge carrier annihilation, effectively mitigating the photocorrosion of g-C_3_N_4_ (Du et al., 2021). Consequently, in the ternary g-C_3_N_4_/MnO_2_/GO photocatalytic system, a pronounced enhancement is observed in the photolytic breakdown of tetracycline hydrochloride (TC).

Given the excellent electron transfer properties exhibited by GO in ASSZ photocatalysts, it is imperative to mention reduced graphene oxide (RGO), which similarly boasts exceptional two-dimensional layered structure and electron transfer capabilities. In a ternary Z-scheme TiO_2_/RGO/g-C_3_N_4_, as prepared by Wu et al. (2017), RGO acts as a conduction shuttle, effectively suppressing recombination in charge carriers and facilitating the Z-scheme charge separation. The introduction of RGO augments the specific surface area of the sample, leading to an increased number of adsorption and photocatalytic sites. Simultaneously, the strong interaction between RGO and g-C_3_N_4_/TiO_2_ results in a notable narrowing of the bandgap and heightened absorption of visible light. The collaborative impacts of RGO contribute to the enhanced photocatalytic decolorization activity through its multifunctional roles. Ibrahim et al. (2020) utilized pulsed laser ablation in liquids to fabricate a Z-scheme photocatalytic TiO_2_/RGO/g-C_3_N_4_ nanocomposite. The photocatalyst exhibited a hydrogen production that was 93-fold greater than that observed with primitive g-C_3_N_4_. In this context, RGO itself does not directly contribute to photocatalytic hydrogen production; its primary role is to play the role of an electron transport shuttle from TiO_2_ to g-C_3_N_4_. Thereby, it significantly promotes the constitution of a Z-scheme photocatalytic heterojunction for electron transfer between TiO_2_ and g-C_3_N_4_, simultaneously increasing contact region and enhancing contact tightness between the two semiconductors, leading to improved surface reactions and adsorption kinetics.

### 2.4 Metallic compound as electron shuttle

When constructing Z-scheme heterojunction photocatalysts through g-C_3_N_4_ combination with other semiconductors, it is advantageous to utilize another metallic compound as an electron conductor to bridge the two semiconductors and promote the establishment of the heterojunction. In the research aimed at enhancing the photocatalytic performance through establishing a heterojunction regarding both Ag_3_PO_4_ and g-C_3_N_4_ composites, Zhu et al. (2020) employed low cost and chemically stable ZnO as a shuttle for electron transfer. In terms of the ternary Z-scheme g-C_3_N_4_/Ag_3_PO_4_/ZnO, within a catalyst dosage of 0.6 g/L, a wastewater dosage of 30 mg/L, and a pH level of 6, the TC degradation rate can reach 89.95% under sunlight. It is noteworthy that, during the cyclic experiments for photocatalytic stability assessment, the efficiency of the ternary photocatalyst Ag_3_PO_4_/g-C_3_N_4_/ZnO remained almost unchanged after four cycles, while Ag_3_PO_4_ exhibited a significant decrease in performance after four cycles. This can be attributed to Ag_3_PO_4_, which, under visible light, generates photogenerated electrons that reduce Ag^+^ to Ag^0^. Furthermore, a portion of Ag_3_PO_4_ may decompose into Ag_2_O, resulting in the loss of Ag_3_PO_4_. However, in the existence of ZnO as an electron shuttle, it can effectively transfer e^−^ from Ag_3_PO_4_ CB to g-C_3_N_4_ VB, establishing a Z-scheme electron transmission. The researchers calculated the VB and CB positions of each semiconductor by combining the results of Mott-Schottky and UV-Vis DRS experiments and found a possible pathway for the generation of •O_2_^−^ to propose the mechanism mentioned above. This boosts the capability in separating photogenerated charge carriers, consequently strengthening photocatalytic performance and reducing photocorrosion in Ag_3_PO_4_. Another set of Fe_3_N particles possessing metallic transport nature similarly performs the role of pathways for charge transfer in the ternary Z-scheme photocatalyst Fe_3_N/Fe_2_O_3_/g-C_3_N_4_, promoting electrons migrating between g-C_3_N_4_ and Fe_2_O_3_, thereby enhancing the photocatalytic decolorization of Rh B and the ability for photocatalytic reduction of CO_2_ (Padervand et al., 2021). The synergistic effects among these three different compounds can effectively improve the efficacy of the photocatalyst.

## 3 Direct ternary Z-scheme photocatalysts

### 3.1 Single Z-scheme photocatalytic configuration

With the continuous exploration and research of photocatalytic systems with Z-scheme photocatalytic configuration, a DZ photocatalysis setup that does not require electron shuttle has begun to emerge. Since Wang et al. (2009a) stated the discovery that two semiconductor materials in intimate contact can also achieve electron-hole transfer mechanism in a Z-scheme configuration, there has been an increasing focus on DZ photocatalytic configuration. In a DZ photocatalysis setup, the establishment of a single Z-scheme heterojunction requires only a pair of distinct semiconductors featuring harmonized electronic band configurations. In such a ternary system, the component not involved in the advancement of the Z-scheme heterojunction will play additional distinctive roles.

#### 3.1.1 Single Z-scheme photocatalytic configuration with noble metals

In direct ternary single Z-scheme photocatalysts involving noble metals, while the noble metals do not directly participate in the Z-scheme transfer mechanisms, their presence induces special effects such as Schottky contacts and Surface Plasmon Resonance (SPR). These effects effectively boost the photocatalytic performance. Furthermore, the excellent metal storage and transport capabilities of noble metals also contribute to the augmentation of photocatalytic efficiency. For instance, Liu et al. (2021) fabricated a ternary Z-scheme contact g-C_3_N_4_/NiTiO_3_/Au nanofibers, about 50–60 nm g-C_3_N_4_ nanolayers uniformly formed on the surface NiTiO_3_ nanofibers, establishing uniform core-shell surface contacts. The uniform and closely packed large-area contact between NiTiO_3_ and g-C_3_N_4_ is highly advantageous for facilitating charge transfer in the Z-scheme interface. Building upon their prior research, such interfaces allow for the creation of orderly interfacial built-in electric fields (Tao et al., 2020). The interfacial electric fields resulting from the charge disparity between NiTiO_3_ and g-C_3_N_4_ cause an upward bending in g-C_3_N_4_ at the interface contact region and a downward bending in NiTiO_3_ at the interface contact area. Such contacts represent a typical Z-scheme electron transfer pathway, thus enhancing charge separation successfully. Interestingly, the addition of Au onto the binary NiTiO_3_/g-C_3_N_4_ nanofibers led to a substantial advancement in photocatalytic capability. While Au does not participate in Z-scheme migration, the incorporation of Au nanoparticles induces fluctuations concerning the surface potential range of g-C_3_N_4_ nanolayer as the result of Schottky junction effect. Such Schottky surface contacts create a new charge transfer pathway, contributing to enhanced charge separation. In this ternary Z-scheme photocatalytic configuration, the collaborative effect of Schottky junction and Z-scheme results in superior photocatalytic activity when compared to individual and binary photocatalysts.

Similarly, Ye et al. (2019) also constructed ternary Ag/Bi_4_O_7_/g-C_3_N_4_ nanosheets, which exhibit significantly higher performance in photocatalysis in contrast to unmodified g-C_3_N_4_, Bi_4_O_7,_ and Bi_4_O_7_/g-C_3_N_4_ nanometric sheets. In Bi_4_O_7_/g-C_3_N_4_, a Z-scheme photocatalysis setup is formed, effectively promoting powerful separation of charges while retaining photoexcited electron-hole pairs with strong oxidation-reduction capabilities. Furthermore, the superior electrical conductance of Ag facilitated electron transfer, further enhancing charge separation. During the photocatalytic remediation of hexavalent chromium (Cr(VI)), the SPR effect by Ag nanoparticles holds a key facilitating position. Within the SPR phenomenon, plasmonic electrons generated by Ag nanoparticles can be transported to g-C_3_N_4_, and the existence of Schottky barriers at interface interaction of Ag nanoparticles and g-C_3_N_4_ nanosheets contributes to electron accumulation on g-C_3_N_4_. In another ternary g-C_3_N_4_/Ag/ZnO photocatalysts prepared by Sher et al. (2021), g-C_3_N_4_ and ZnO formed a heterojunction with Z-scheme configuration, and Ag was decorated on the exterior of ZnO as a facilitator to strengthen the separation of photoelectron-hole pairs and improve electron transport.

In ternary systems where two semiconductors form a DZ heterojunction, the third component that does not participate in the Z-scheme pathway typically introduces novel performance enhancements. El-Sheshtawy et al. (2019) prepared a Z-scheme photocatalysts with g-C_3_N_4_ and V_2_O_5_, and the addition of the noble metal Ag facilitated electron storage, leading to a beneficial post-degradation effect. The trace concentration of Ag nanoparticles, decorated upon the outer layer of V_2_O_5_/g-C_3_N_4_ employing a sol-gel photodeposition approach to create the ternary Z-scheme Ag/V_2_O_5_/g-C_3_N_4_. Under sunlight illumination, the Z-scheme Ag/V_2_O_5_/g-C_3_N_4_ photocatalyst can reduce *p*-nitrophenol within 8 min. In the absence of sunlight, complete *p*-nitrophenol reduction is also achieved within 60 min. For the purpose of investigating the post-illumination efficiency of Ag/V_2_O_5/_g-C_3_N_4_, the photocatalyst was exposed to sunlight for 30 min and then introduced into a dark environment with Cr^+6^ solution, resulting in a 33% reduction in Cr^+6^ concentration within 60 min. This particular phenomenon is attributed to the inherent electron storage capability of the loaded Ag nanoparticles. The rapid photocatalytic degradation efficiency of *p*-nitrophenol benefits from the Z-scheme design established on V_2_O_5_/g-C_3_N_4_, then the addition of Ag effectively enhances photocatalytic activity. Furthermore, its contribution to excellent pollutant degradation under dark reaction conditions provides an advantage for water treatment by the photocatalyst under different conditions.

#### 3.1.2 Single Z-scheme photocatalytic configuration without noble metals

In the investigation of ternary DZ g-C_3_N_4_ based photocatalysts, not only noble metals can facilitate electron transfer and synergistic effects to enhance photocatalytic efficiency in the third component that does not directly participate in Z-scheme transport, but metal compounds can also have a significant promoting effect. In ternary photocatalysts composed of CoS, CdS, and g-C_3_N_4_, large inner space g-C_3_N_4_ hollow nanosphere was prepared by utilizing the tunable microstructure in g-C_3_N_4_. Subsequently, CdS was affixed to surface area of g-C_3_N_4_, followed by placing of CoS onto the hollow spherical g-C_3_N_4_ (Zhang et al., 2023). Based on the Mott-Schottky analysis, a Z-scheme forms amidst CdS and g-C_3_N_4_ due to their suitable energy band configuration. Furthermore, the CoS attached on the surface of g-C_3_N_4_ represents an electron accumulator, expediting the rate of transfer for photogenerated electrons. Electrons in the g-C_3_N_4_ CB are shifted to CoS, where they interact with H^+^ to form H_2_, thereby significantly enhancing hydrogen production performance.

As early as 2013, Yu et al. (2013) presented an establishment of a successful DZ semiconductor junction photocatalytic structure amidst g-C_3_N_4_ and TiO_2_. Subsequent research efforts have continuously advanced this DZ heterojunction. Wolde et al. (2022) proposed a ternary g-C_3_N_4_/MgO/TiO_2_. Positioned at the junction of g-C_3_N_4_/TiO_2_, a Z-scheme configuration is formed, and the loading of MgO onto TiO_2_ introduces Ti^3+^ defects and oxygen vacancy defects. The Z-scheme architecture formed within the boundary of TiO_2_ and g-C_3_N_4_ interface has effectually promoted the photoelectron-holes separation, thereby enhancing photocatalytic performance. The incorporation of MgO further improves charge separation efficiency, owing to the leading of oxygen vacancy-related surface defects and Ti^3+^ through the interfacial interplay of MgO and TiO_2_. The photocatalytic efficiency of ternary heterostructure g-C_3_N_4_/MgO/TiO_2_ has improved threefold when compared with TiO_2_/g-C_3_N_4_ and MgO/TiO_2_ binary systems.

### 3.2 Dual Z-scheme photocatalytic configuration

**FIGURE 4:**
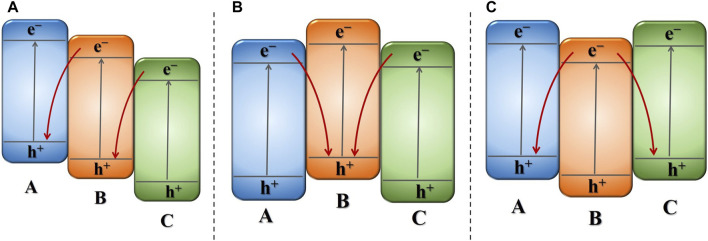
Schematic illustration of different types of direct dual Z-schemes photocatalytic system: **(A)** middle semiconductor acting as an a shuttle, **(B)** h^+^ recombining with e^−^ in middle semiconductor, **(C)** e^−^ recombining with h^+^ associated with two-sided semiconductor.

When ternary semiconductor materials possess matching band structures, they can form a dual DZ-scheme structure with extensive capacity for photon capture and promoted electron migration. Furthermore, ternary Z-scheme photocatalysts with appropriate band structures enable more effective charge transfer/separation than binary Z-scheme photocatalysts with single Z-scheme pathway (Liu et al., 2018; Katsumata et al., 2022). In the dual DZ-scheme g-C_3_N_4_-based ternary photocatalytic configuration, as no noble metal is loaded, there is no light shielding effect on the semiconductors, allowing all semiconductors to effectively absorb light energy and generate charge carriers. Based on distinct pathways of electron and hole recombination in direct dual Z-scheme photocatalytic configuration, they can be classified as three kinds of dual DZs. As shown in Figure 4, the three semiconductors constituting the dual Z-scheme photocatalytic configuration are labeled as A, B, and C. The middle semiconductor situated within the dual Z-scheme photocatalytic configuration, created by the tight interaction of three semiconductors, serves as the basis for categorization. The first type involves the middle semiconductor acting as a shuttle, where e^−^ within the CB of C recombination with the h^+^ within the VB of B, and the e^−^in the CB of B recombination with the h^+^ within the VB of A. The second type involves the h^+^ in the VB of the B recombining with the e^−^ in the CB of A and C. The third type involves e^−^ within the CB of the B transferring and recombining with h^+^ associated with the VB of A and C.

#### 3.2.1 Middle semiconductor acting as an shuttle

Facilitate the flow of charge carriers and oxidation-reduction capability of Z-scheme heterojunctions based on g-C_3_N_4_, it has been an effective approach to seek suitable semiconductor materials with appropriate band structures for coupling. Particularly, the combination of three different semiconductors in a dual Z-scheme photocatalysis setup has proven to be more advantageous in achieving this objective. Recently, Sarkar et al. (2022a) presented that a dual Z-scheme g-C_3_N_4_/ZnFe_2_O_4_/Bi_2_S_3_ photocatalyst exhibited higher photodegradation activity of 2,4,6-tricholorophenol (TCP) than any of the pristine materials or binary composites. In this dual Z-scheme photocatalyst, an intimate Z-scheme heterojunction structure is formed in g-C_3_N_4_/ZnFe_2_O_4_, with Bi_2_S_3_ subsequently growing on g-C_3_N_4_/ZnFe_2_O_4_. Energy band information and e^−^/h^+^ transfer pathways were obtained *via* Mott-Schottky analysis and XPS. In this system, electrons move across from the elevated Fermi level to the lower Fermi level, causing e^−^ within CB of ZnFe_2_O_4_ annihilate with the h^+^ in VB of g-C_3_N_4_. Subsequently, e^−^ transfer from Bi_2_S_3_ to ZnFe_2_O_4_, resulting in ZnFe_2_O_4_ acting as an electron shuttle. This leads to the accumulation of e^−^in g-C_3_N_4_ and h^+^ in Bi_2_S_3_. Based on this, effective charge separation is achieved, preserving the highest oxidative and reductive capabilities of electrons and holes. Such a ternary dual Z-scheme photocatalysts substantially boosts the efficiency of charge separation and enhances the capacity to absorb visible light.

This structure was not only proposed in g-C_3_N_4_/ZnFe_2_O_4_/Bi_2_S_3_, but the same researchers Sarkar et al. also offered the same Z-scheme transport process in g-C_3_N_4_/CuFe_2_O_4_/MoS_2_. Analogous with the previously mentioned structure, CuFe_2_O_4_ acts as an electron shuttle in this structure. The same structural concept was not limited to g-C_3_N_4_/ZnFe_2_O_4_/Bi_2_S_3_, Sarkar et al. (2022b) also applied a similar Z-scheme conveyance of charge process in g-C_3_N_4_/CuFe_2_O_4_/MoS_2_. In this dual Z-scheme photocatalytic configuration, CuFe_2_O_4_ serves in the role of an electron shuttle, causing the piling up of e^−^ in g-C_3_N_4_ and h^+^ in MoS_2_, which are later engaged throughout the photocatalytic process. And the same dual Z-scheme structure also been proposed such as g-C_3_N_4_/ZnS/ZnO (Dong et al., 2018), g-C_3_N_4_/MoS_2_/Ag_3_PO_4_ (Tian et al., 2019), g-C_3_N_4_/Zn_2_SnO_4_N/ZnO (Wang M. et al., 2019), g-C_3_N_4_/MoS_2_/ZnO (Madhushree et al., 2022), g-C_3_N_4_/CeO_2_/Bi_2_O_3_ (Devi K R et al., 2020), and g-C_3_N_4_/MoS_2_/TiO_2_ (Jaleel et al., 2021). In those ternary Z-scheme g-C_3_N_4_-based heterojunctions, g-C_3_N_4_ serves as a photocatalyst for reduction. Electrons accumulate in g-C_3_N_4_ and contribute to the photogenerated reduction process. Among the ternary of semiconductors, the one with a CB position not being the highest and a VB position not being the lowest is located in the middle of the dual Z-scheme photocatalytic configuration. This semiconductor acts as an electron shuttle and can include materials like ZnS, MoS_2_, Zn_2_SnO_4_N, and CeO_2_. Then, the third semiconductor with the lowest VB position functions as an oxidation photocatalyst in the dual Z-scheme photocatalytic configuration, and materials like ZnO, Ag_3_PO_4_, Bi_2_O_3_, and TiO_2_ can play this role.

#### 3.2.2 Electron transfer from both sides semiconductor to the middle semiconductor

**FIGURE 5:**
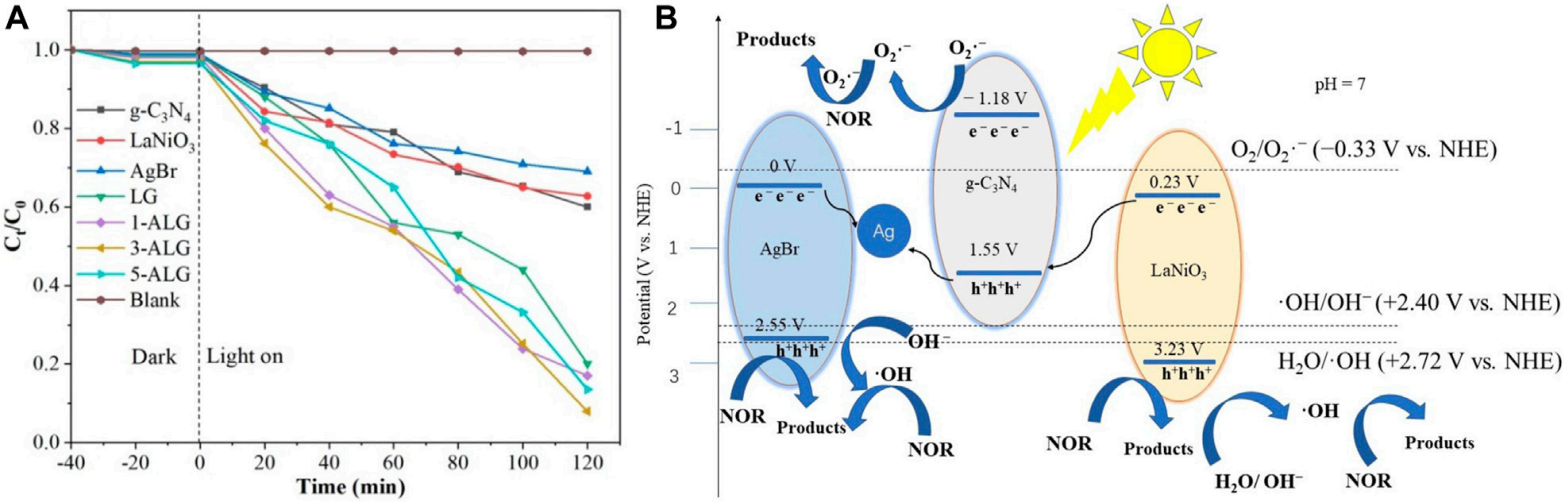
**(A)** Photocatalytic performances for norfloxacin degradation over different photocatalysts. **(B)** Proposed mechanism of as-prepared AgBr/g-C_3_N_4_/LaNiO_3_. Reproduced with permission from Zhang J. et al. (2020), Copyright 2020 MDPI.

Since the semiconductor materials exhibit distinct energy band structures, when selecting two semiconductors with appropriate energy band structures to integrate with g-C_3_N_4_ to build a dual Z-scheme heterojunction, a structure is formed in which g-C_3_N_4_ is positioned centralized within dual Z-scheme transport channel. In this structure, and the e^−^ in the CB of the other two semiconductors merges with the h^+^ in VB of g-C_3_N_4_. Saravanakumar and Park (2021) designed a g-C_3_N_4_/BiFeO_3_/LaFeO_3_ dual Z-scheme photocatalyst using a wet chemical process, exposed to visible light for a time span of 60 min, the photocatalytic reactivity of CIP achieved a degradation rate of 98.6%. The outcomes of experiments involving radical trapping and measurements of ESR in this system proved the formation of both •O^2−^ and •OH reactive species over the span of the photodegradation reaction, thereby enhancing the oxidation capacity of the photocatalyst. However, the energy levels in CB of BiFeO_3_ and LaFeO_3_ are insufficiently elevated to generate •O^2−^ radicals, while the VB potentials of g-C_3_N_4_ are inadequate for •OH radicals to be produced. From level of energy bands computations and empirical data, proved charge migration pathway adheres a dual Z-scheme, not the traditional type-II photocatalytic mechanism. With such double Z-scheme photocatalytic configuration, g-C_3_N_4_ possesses a more negative CB edge compared to LaFeO_3_ and BiFeO_3_, while the VB of both BiFeO_3_ and LaFeO_3_ are more positive than that of g-C_3_N_4_. These three semiconductors constitute a symmetric double Z-scheme heterojunction. In this ternary heterostructure, they not only promote charge transfer but also maintain strong reduction/oxidation abilities. The same dual Z-scheme system g-C_3_N_4_/AgBr/LaNiO_3_, also proposed by Zhang J. et al. (2020), employing a simple ultrasound-assisted hydrothermal strategy, AgBr nanoparticles and LaNiO_3_ nanoballs were adhered to the external side of g-C_3_N_4_ nanosheets. As illustrated in Figure 5A, the performance of ternary Z-scheme AgBr/g-C_3_N_4_/LaNiO_3_ exhibited remarkable enhancement, and 92% of the norfloxacin was degraded within 120 min, which surpasses the degradation rate achieved by bare g-C_3_N_4_ (40%), AgBr (38%), LaNiO_3_ (31%), and g-C_3_N_4_/LaNiO_3_ (80%). It is noteworthy that, within this ternary Z-scheme photocatalyst, the Ag ion in AgBr can easily undergo reduction to form metallic Ag under illumination. The resulting metallic Ag serves as an effective electron transfer center, promoting the efficient separation of photocatalytic charge pairs throughout the photocatalytic reaction, schematic is illustrated in Figure 5B. In this dual Z-scheme structure, e^−^ produced in the CB of AgBr and LaNiO_3_ will undergo recombination with h^+^ within the VB region of g-C_3_N_4_. Subsequently, the e^−^ remaining situated at the CB states of g-C_3_N_4_ and the h^+^ within VB of AgBr and LaNiO_3_ actively contribute to the photocatalysis. In the vein of this ternary Z-scheme structure, g-C_3_N_4_ demonstrates the highest CB position located in the center, while the two semiconductors have lower VB positions situated on both sides of the symmetric photocatalytic system employing a Z-scheme configuration. These symmetric Z-scheme were also proposed in WO_3_/g-C_3_N_4_/Bi_2_O_3_ (Jiang L. et al., 2018), Bi_2_O_3_/g-C_3_N_4_/Ag_6_Si_2_O_7_ (Zhao H. et al., 2020), AgBr/g-C_3_N_4_/BiPO_4_ (Li Y. et al., 2021).

In this ternary Z-scheme transfer channel system, where electrons are transferred from the semiconductors on both sides to the middle semiconductor, if g-C_3_N_4_ does not possess the most negative CB potentials among the three semiconductors, the semiconductor with the most negative CB potentials is located in the central position. Kang et al. (2020) proposed a dual Z-scheme photocatalysts MoS_2_/Bi_24_O_31_C_l10_/g-C_3_N_4_, which can eradicate 97.5% of TC within 50 min with illumination by visible light. In this ternary Z-scheme g-C_3_N_4_-based photocatalytic configuration, the CB potential of Bi_24_O_31_C_l10_ shows a greater negative potential than g-C_3_N_4_, positioning Bi_24_O_31_C_l10_ in the central position. When exposed to light, photoinduced charge carriers are generated in semiconductor, electrons originating from the CB in g-C_3_N_4_ and MoS_2_ migrate across to the VB of Bi_24_O_31_C_l10_, resulting in their recombination. In this dual Z-scheme photocatalytic configuration, it also retains an increased quantity of carriers with enhanced redox capability. It has also been demonstrated that this photocatalytic mechanism exists in g-C_3_N_4_/Co_3_O_4_/CoO, with CoO possessing the most negative CB potential and being placed in the central position between Co_3_O_4_ and g-C_3_N_4_ (Zheng and Zhang, 2019). The g-C_3_N_4_/Co_3_O_4_/CoO ternary heterojunction exhibits excellent photocatalytic conversion towards diminution of nitrobenzene (NB) and tetracycline (TC) photodegradation efficiency, as well as excellent magnetic separation properties.

#### 3.2.3 Electron transfer from the middle semiconductor to both sides semiconductor

In ternary dual Z-scheme photocatalysis on g-C_3_N_4_, another electron transport structure is formed among the three semiconductors, where electrons located within the middle semiconductor recombine with holes on both sides of the semiconductor. Generally, g-C_3_N_4_, along with another semiconductor, typically occupies a higher CB position in this Z-scheme photocatalytic configuration and exhibits strong reduction capabilities. These can be considered as reduction photocatalysts positioned on the two sides. The semiconductor possessing a lower VB position is situated in the center and can be considered as an oxidation photocatalysts. Yin et al. (2021) synthesize a ternary g-C_3_N_4_/AgBr/β-Ag_2_WO_4_ dual Z-scheme photocatalysts in the decomposition of organic contaminants. The dual Z-scheme charge carrier transfer pathway, where e^−^ within CB in β-Ag_2_WO_4_ merge with h^+^ associated with the VB of g-C_3_N_4_ and AgBr through recombination, has improved photocatalytic degradation performance. Photocatalytic reduction reactions take place at the higher CB positions of g-C_3_N_4_ and AgBr, while the oxidation reactions occur at the VB of β-Ag_2_WO_4_ at lower positions. The unique transport pathway for photogenerated carriers significantly contributes to the enhancement of catalytic activity. Notably, α-Ag_2_WO_4_, which shares the same chemical composition as β-Ag_2_WO_4_ but possesses a different crystal structure, also forms a photocatalyst featuring dual Z-scheme incorporating g-C_3_N_4_. Ayappan and Mani (2023) manufactured a dual Z-scheme g-C_3_N_4_/α-Ag_2_WO_4_/Bi_2_S_3_ photocatalyst. In this dual Z-scheme photocatalyst, α-Ag_2_WO_4_ also occupies the central position and serves as an oxidation photocatalyst. Some other studies with the same dual Z-scheme photocatalytic mechanism such as g-C_3_N_4_/WO_3_/AgI (Tang et al., 2020) and g-C_3_N_4_/Bi_2_WO_6_/AgI (Xue et al., 2019) have been proposed in recent years.

## 4 Application of ternary Z-scheme g-C3N4 based photocatalyst

### 4.1 Removal of pollutant in wastewater

In the past few years, as environmental water contamination has become more and more severe, the use of oxidation advanced treatment has received widespread attention. Among the various effective methods, photocatalysis technology garners significant attention because of its high efficiency, stability, and its efficiency in capturing sunlight effectively (Regmi et al., 2018; Yang et al., 2021b). Ternary Z-scheme photocatalysis technology based on g-C_3_N_4_ has been extensively utilized for environmental water pollution treatment, with one of its primary applications being the photocatalytic decolorization of organic dyes in sewage. A Z-scheme MoS_2_/g-C_3_N_4_/ZnO (Madhushree et al., 2022) ternary photocatalyst employed for the photocatalytic decolorization of malachite green (MG), and a Z-scheme photocatalytic configuration g-C_3_N_4_/C/S-g-C_3_N_4_ (Yang Y. et al., 2021) system for the photocatalytic decolorization of rhodamine-B (RhB), congo red (CR), and methylene blue (MB). Methyl orange (MO) can also be successfully broken down with the attendance of rGO/Fe_2_O_3_/g-C_3_N_4_ (Park et al., 2022) as photocatalyst exposed to solar light irradiation. For the purpose of examining the impact of dye density, catalyst amount to be taken, and pH on photocatalytic decolorization of organic dyes, Padervand et al. (2021) researched the decolorization efficiency of RhB using the Fe_3_N/Fe_2_O_3_/g-C_3_N_4_photocatalyst under various different conditions. The optimum photocatalytic decolorization efficiency was accomplished with a catalyst input of 0.04 g, the pH value was 3.5, and the dye concentration was 5 ppm.

The residues of antibiotics, which are difficult to biodegrade and possess long-term toxicity, persist in water bodies and exert an increasing impact on the environment with the rising usage of antibiotics. The significant existence of antibiotic contamination in the environment possesses the capacity to induce microbial mutation and adaptation, adding to the problem of animal resistance to antibiotics (Huo et al., 2016; Zhao et al., 2021). Consequently, the issue of treating antibiotic residues in water has gained growing attention. Among the various methods available, Z-scheme g-C_3_N_4_-based photocatalysis stands out as a promising tactic for overcoming this challenge. In the study by Ayappan and Mani (2023), a ternary Z-scheme g-C_3_N_4_/Bi_2_S_3_/α-Ag_2_WO_4_ was employed for the degradation of tetracycline (TC). This ternary Z-scheme photocatalyst achieved a 95.02% degradation of TC within 120 min. Interestingly, they used the solution resulting from the photocatalyst degradation treatment to cultivate mung bean sprouts and test their biotoxicity. The results demonstrated that mung bean sprouts germinated in the photocatalytically degraded TC solution. This effectively proves that, under sunlight irradiation, Bi_2_S_3_/g-C_3_N_4_/α-Ag_2_WO_4_ successfully decomposes TC and reduces its toxicity to organisms. In the investigation of Kang et al. (2020), 97.5% of TC was effectively removed within 50 min when illuminated with light in the perceptible range. The Z-scheme photocatalysis on g-C_3_N_4_not only effectively degrades TC (Xue et al., 2019; Zheng and Zhang, 2019; Kang et al., 2020; Yu et al., 2020; Du et al., 2021) but has also demonstrated excellent photocatalytic degradation effects on tetracycline hydrochloride (Asadzadeh-Khaneghah et al., 2021), sulfamethoxazole (Ren et al., 2019), metronidazole (Jo and Natarajan, 2016), norfloxacin (Zhang J. et al., 2020), and ciprofloxacin (Deng et al., 2018; Saravanakumar and Park, 2021; Sarkar et al., 2022b).

Furthermore, addressing heavy metal contamination in wastewater is a formidable challenge. For instance, the improper treatment of industrial wastewater can introduce a significant risk to organisms, particularly regarding Cr(VI), necessitating urgent attention. In the study by Ghafoor et al. (2019), Ag/TiO_2_/g-C_3_N_4_ photocatalyst has demonstrated remarkable effectiveness in the photo-reduction of Cr(VI). This photocatalyst is capable of converting all Cr(VI) to Cr(III) in 150 min with solar light irradiation. The ternary g-C_3_N_4_/Ag/Bi_4_O_7_ nanosheets prepared by Ye et al. (2019) have also been implemented for photocatalytically reducing aqueous Cr(VI). The specific surface area of g-C_3_N_4_ was significantly enhanced through thermal spalling, with the distribution of Bi_4_O_7_ and Ag on the surface of g-C_3_N_4_ nanosheets. Subjected to illumination within the visible spectrum, the top-performing photocatalyst reached a reduction of almost 98% in Cr(VI) concentration within 60 min. In another ternary Z-scheme photocatalytic configuration, Ag/g-C_3_N_4_/V_2_O_5_, the photogenerated reduction of Cr(VI) can also be achieved through photocatalytic reactions (El-Sheshtawy et al., 2019).

### 4.2 Water splitting

Utilizing photocatalysts for overall water cracking is deemed an affordable procedure for sunlight harvesting to yield hydrogen and oxygen. However, the selection of the photocatalyst in this process is limited by the requirement that it must have a suitable bandgap position, be capable of straddling the redox potential of water decomposition into hydrogen and oxygen, and meet the condition of appropriate surface reaction kinetics and good stability (Moniz et al., 2015; Chen S. et al., 2017; Zhao W. et al., 2020). Theoretically, g-C_3_N_4_ satisfies the aforementioned criteria and represents an ideal photocatalyst for facilitating the water dissociation process for the production of hydrogen and oxygen. Nevertheless, practical uses of pristine g-C_3_N_4_ encounter issues for instance the high-speed recombination of photoinduced electron-hole pairs, which is related to its inefficiency in the water splitting process. Consequently, research endeavors focused on boosting effectiveness of g-C_3_N_4_ for water cleavage applications have witnessed continuous and concerted efforts over the past several years (Cao et al., 2015; Gao et al., 2022). To construct Z-scheme heterojunction photocatalysts based on g-C_3_N_4_ is an effective approach.

The ternary Z-scheme photocatalysis on g-C_3_N_4_ photocatalysts demonstrated excellent photocatalytic capabilities in water splitting process for hydrogen evolution. Zhang et al. (2023) synthesized a CoS/CdS/g-C_3_N_4_ Z-scheme configuration, which displayed a significantly advanced hydrogen evolution level of 2,866 μmol⋅g^−1^⋅h^−1^. The hollow spherical g-C_3_N_4_ exhibits a larger specific surface area compared to bulk g-C_3_N_4_, thereby enhancing the photocatalytic reaction. This rate here is 20-fold increase compared to hollow spherical g-C_3_N_4_ and 1.4 times more than CdS/g-C_3_N_4_ in their as-prepared samples. Jo and Selvam (2017) presented findings on Z-scheme photocatalytic configuration g-C_3_N_4_/CdS with RGO for the photochemical hydrogen evolution from lactic acid-infused water amidst light in the visible spectrum. The rate of hydrogen creation achieved 676.5 μmol⋅g^−1^⋅h^−1^, Reflecting a36.5% apparent quantum efficiency (AQE) performance. Furthermore, the system demonstrated robust photostability. Ibrahim et al. (2020) enhanced the success rate of hydrogen yield through water cracking by incorporating TiO_2_ nanotubes, RGOand g-C_3_N_4_ nanosheets to fabricate a ternary Z-scheme photocatalyst g-C_3_N_4_/RGO/TiO_2_. The g-C_3_N_4_/RGO/TiO_2_ system established a notable rate at 32 mmol⋅g^−1^⋅h^−1^ which hydrogen is produced, surpassing the rates observed for pristine g-C_3_N_4_, TiO_2_, and TiO_2_/RGO by approximately 93, 3.8, and 2.6 times, respectively.

In contrast to photocatalytic water splitting aimed at hydrogen evolution, the procedure for oxygen generation by water cracking presents more significant challenges. The efficiencies of water splitting for oxygen production are comparatively lower than those for hydrogen production, primarily due to the sluggish kinetics that need to be overcome and the substantial overpotential required for the evolution reaction (Li et al., 2022). The dual DZ photocatalytic configuration has witnessed outstanding advances in extremely efficient water splitting for oxygen production. Tian and co-workers (Tian et al., 2019) presented a Z-scheme photocatalytic configuration comprising g-C_3_N_4_/MoS_2_/Ag_3_PO_4_, achieving the highest oxygen generation rate recorded at 232.1 μmol L^−1^⋅g^−1^⋅h^−1^. In comparison, the other components, Ag_3_PO_4_, Ag_3_PO_4_/MoS_2_, and Ag_3_PO_4_/g-C_3_N_4_, exhibited lower oxygen generation rates of 45.9 μmolL^−1^⋅g^−1^⋅h^−1^, 189.5 μmolL^−1^⋅g^−1^⋅h^−1^, and 198.4 μmolL^−1^⋅g^−1^⋅h^−1^, respectively. Another g-C_3_N_4_-based dual Z-scheme configuration, ternary g-C_3_N_4_/Ag_2_MoO_4_/Ag_3_PO_4_ composite, also exhibited the highest oxygen production rate recorded at 924.6 μmolL^−1^⋅g^−1^⋅h^−1^, surpassing that of Ag_3_PO_4_, Ag_2_MoO_4_ and Ag_2_MoO_4_/Ag_3_PO_4_ (Liu et al., 2018). Si et al. (2018) presented a DZ Ag_3_PO_4_/graphdiyne/g-C_3_N_4_ component compound, demonstrating improved oxygen formation with a speed of 753.1 μmol⋅g^−1^⋅h^−1^. In this photocatalyst, graphdiyne serves as a conductive electron shuttle among Ag_3_PO_4_ and g-C_3_N_4_ in the Z-scheme photocatalysis setup. Additionally, graphdiyne also acts as a foundation for maintaining Ag_3_PO_4_, thereby enhancing O_2_ evolution.

### 4.3 CO_2_ reduction

In response to the concern of global warming caused by carbon dioxide (CO_2_) emissions, a greenhouse effect contributor, the photocatalytic synthesis of high-value chemicals from carbon dioxide, for instance, hydrocarbon fuels, has emerged as an encouraging green technology. This strategy, acting as getting double mileage out of one effort, not only offers a solution to the global warming problem but also addresses the challenges associated with energy and fuel shortages (Kumar et al., 2018; Wang et al., 2020; Jia et al., 2023). However, the fully oxidized CO_2_ is extremely stable, the CO_2_ reduction needs huge bond dissociation energy for C-O bond dissociation and C-H bond forming. By way of contrast, the mechanism and process of CO_2_ photoreduction is complicated, products are diverse. Nevertheless, the fully oxidized CO_2_ results in exceptional stability. CO_2_ reduction necessitates a substantial bond dissociation energy for the dissociation of C-O bonds and the creation of C-H bonds (Xu D. et al., 2018). Simultaneously, the complexity of CO_2_ photoreduction lies in its intricate mechanisms and processes, yielding a diverse array of products (Ong et al., 2016; Tasbihi et al., 2018). Among the numerous photocatalysts employed for CO_2_ reduction, the distinctive combination of an applicable bandgap and aligned conduction and valence band positions makes g-C_3_N_4_ stand out. Consequently, Z-scheme heterojunction photocatalysts on g-C_3_N_4_ prove highly effective in overcoming challenges associated with CO_2_ photoreduction.

Madhusudan et al. (2021) synthesized an outstandingly effective ternary Zn_0.5_Cd_0.5_S/Au@g-C_3_N_4_ Z-scheme photocatalysis setup for transforming CO_2_ into methanol (CH_3_OH), with formaldehyde (HCHO) and methane (CH_4_) observed as minor by-products. The rate of photocatalyzed reduction CO_2_ into CH_3_OH achieved 1.31 μmol ⋅h^−1^⋅g ^−1^, demonstrating a 32.7 times increase over g-C_3_N_4_ (0.04 μmol ⋅h^−1^⋅g ^−1^) and 43.6 -fold boost in comparison with Zn_0.5_Cd_0.5_S (0.03 μmol ⋅h^−1^⋅ g ^−1^). Xu D. et al. (2018) stated the evolution of a DZ Ag_2_CrO_4_/GO/g-C_3_N_4_ photocatalyst in the field of reduction CO_2_ into CH_3_OH, with a minor production of CH_4_. This ternary photocatalytic configuration based on g-C_3_N_4_ demonstrated CO_2_ conversion efficiency reaching 1.03 μmol g^−1^, semonstrating a TOF of 0.30 h^–1^ during the initial 3 h of illumination across the entire spectrum.

Photocatalyst conversion of CO_2_ to renewable carbonaceous fuels can not only produce CH_3_OH but also generate carbon monoxide (CO) and methane (CH_4_) as a common green fuel product. Raza et al. (2020) prepared a g-C_3_N_4_/Pt/Cu_2_ZnSnS_4_ for transforming CO_2_ into high-value carbonaceous fuels. In this Z-scheme photocatalyst, the CO production capacity reached 242.3 μmol⋅h^−1^⋅ g ^−1^, and the CH_4_ yield rate reached 7.961 μmol ⋅h^−1^⋅ g ^−1^ with illumination in the visible spectrum. Padervand et al. (2021) also observed the production of 8.03 μmol ⋅h^−1^⋅ g ^−1^ and 1.6 μmol ⋅h^−1^⋅ g ^−1^ of CO and CH_4_, respectively, from the ternary Z-scheme g-C_3_N_4_/Fe_3_N/Fe_2_O_3_ during CO_2_ conversion with H_2_O vapor.

## 5 Conclusion and perspective

Semiconductor photocatalytic technology emerges as an exceptionally encouraging approach for tackling issues associated with environmental pollution and the shortage of energy. The robust breakthrough of this technology, alongside the exploration of efficient photocatalysts, has garnered considerable interest in the past few years. Among the various options for semiconductor photocatalysts, g-C_3_N_4_, featuring a small energy gap with well-positioned CB and VB, exhaustively probed as an outstandingly advantageous photocatalyst for visible-light applications. This is in accordance with its advantageous features, including non-toxicity, low cost, simplicity in preparation, environmentally friendly, and robust stability. However, the investigation into enhancing the performance of g-C_3_N_4_, is driven by its low photocatalytic efficiency stemming from a restricted surface area and an elevated rate of recombination for photogenerated carriers. Constructing a Z-scheme photocatalysis has validated its efficacy in addressing the challenging issues associated with pristine g-C_3_N_4_. In this review, we converse about the design and assembly of various ternary photocatalytic systems based on g-C_3_N_4_, encompassing ASS and DZ heterojunctions. We highlight the attractive properties of these systems and provide a brief summary of the utilization of ternary Z-scheme photocatalytic configuration based on g-C_3_N_4_.

Although ternary Z-scheme photocatalytic configuration based on g-C_3_N_4_ has demonstrated optimized photocatalytic performance, there are still obstacles and challenges that urgently need to be addressed. The following proposals are put forth for future studies:

(1) Modify and engineer an appropriate geometric structure. In Z-scheme heterojunction photocatalytic system, the interfacial interactions between semiconductors contribute significantly to determining photocatalytic capabilities. Therefore, the comprehensive development of nanostructured Z-scheme g-C_3_N_4_-based composites is instrumental in enhancing photocatalytic efficiency. This improvement can be ascribed not just to the formation and isolation of photoexcited electron-hole pairs but also to the facilitation of their transmission to the surface. Simultaneously, a semiconductor in a Z-scheme heterojunction with an appropriately designed structure and surface texture proves exceptionally well-suited for the adsorption of reactants and products, thereby maximizing the effectiveness of the photocatalytic process. In the pursuit of increasing the outer surface of g-C_3_N_4_, numerous studies have investigated its morphology and structure. The distinctive layered two-dimensional (2D) geometry of g-C_3_N_4_ has spurred extensive research dedicated to the assembly of 2D g-C_3_N_4_ nanosheets. Not to mention the synthesis of g-C_3_N_4_ nanosheets, recent research has emphasized the crafting of sponge-like g-C_3_N_4_ and cavernous g-C_3_N_4_ nanospheres. These structural studies and designs have been demonstrated as efficacious techniques for enhancing photocatalytic performance. Hence, there is considerable merit in furthering the expedition preparation of Z-scheme photocatalysis on g-C_3_N_4_, focusing on achieving a larger specific surface region and porous morphology. This is attainable by exploiting the easily modifiable shape arrangement of g-C_3_N_4_, with the aim of boosting the effectiveness of photocatalysis.
(2) In-depth investigation of the mechanism in Z-scheme transport. A comprehensive exploration of the Z-scheme transport procedure is indispensable for the effective enhancement and design of Z-scheme photocatalysis on g-C_3_N_4_. Therefore, to advance these improvements, a detailed investigation is important to characterize the mechanism and charge transport processes associated with Z-scheme photocatalytic configuration. While most studies on the mechanisms of Z-scheme photocatalytic configuration on g-C_3_N_4_ have been confined to verification through photocatalytic degradation experiments with the addition of radical scavengers, more thorough and specific investigations are notably absent. Some studies have employed a combination of analysis through X-ray photoelectron spectroscopy (XPS), photoluminescence (PL), and electron spin resonance (ESR) for a comprehensive analysis of the mechanism. However, the mechanism remains contentious at present. Therefore, the ongoing utilization of advanced characterization instruments is essential to comprehensively elucidate the Z-scheme transport mechanism, benefiting both photocatalyst development and broader applications.
(3) Develop photocatalytic materials that are more favorable for recycling and reuse. In recent years, there has been widespread utilization of Z-scheme g-C_3_N_4_-based photocatalysts, particularly in applications such as hydrogen and oxygen generation from water, as well as in water pollution control. In the above application scenarios, the photocatalysts are mostly required to be dispersed in a liquid environment. However, a significant impediment arises from the conventional methodology employed in the preparation of g-C_3_N_4_ and g-C_3_N_4_-based photocatalysts, involving the generation of a powdered sample through thermal polymerization reactions. This common approach, while widely adopted, presents inherent limitations in terms of convenience for recycling and reuse during practical applications. This hinders the widespread use of photocatalysts. Hence, the synthesis of photocatalysts conducive to enhanced recyclability and reusability, involving strategies like the incorporation of magnetic materials or composites with non-powdered semiconductors, holds significant promise for the purpose of extending the use and fostering the growth of Z-scheme photocatalysts on g-C_3_N_4_.
