# Erratum: Recent advances in ternary Z-scheme photocatalysis on graphitic carbon nitride based photocatalysts

**DOI:** 10.3389/fchem.2024.1447771

**Published:** 2024-07-02

**Authors:** Frontiers Production Office

**Affiliations:** Frontiers Media SA, Lausanne, Switzerland

**Keywords:** G-C_3_N_4_, ternary composite photocatalysts, all-solid-state ternary Z-scheme, direct ternary Z-scheme, application

Due to a production error, in **Table 1**, column 1, the PS I (N) value was given as g-C_34_ instead of g-C_3_N_4_. The corrected table appears below.

**TABLE 1:** Recent progress in g-C_3_N_4_-based ASS ternary Z-scheme photocatalysis with different electron mediators.

PS I (N)	PS II	Electron shuttle	Light source	Application	Activity	Ref
g-C_3_N_4_	MoS_2_	Ag	300 W Xe lamp (λ > 420 nm)	degradation of RhB	DE = 100% (60 min)	**Lu et al. (2017)**
				H_2_ production	104 μmol h^−1^ g^−1^	
g-C_3_N_4_	BiVO_4_	Ag	300 W Xe lamp (λ > 350 nm)	Degradation of TC	DE = 90.76% (60 min)	**Chen et al. (2017a)**
			300 W Xe lamp (λ > 420 nm)		DE = 82.75% (60 min)	
g-C_3_N_4_	NaTaO_3_	Ag	300 W Xe lamp (λ < 420 nm)	Degradation of TC	DE = 95.47% (60 min)	**Tang et al. (2018)**
			300 W Xe lamp (λ > 420 nm)		DE = 91.48% (60 min)	
g-C_3_N_4_	Bi_3_TaO_7_	Ag	300 W Xe lamp	Degradation of SMZ	DE = 98% (25 min)	**Ren et al. (2019)**
g-C_3_N_4_	Ag_3_PO_4_	Ag	300 W Xe lamp (λ > 420 nm)	Removing of NO	74% (90 min)	Li et al. (2021a)
g-C_3_N_4_	BiVO_4_	Ag	300 W Xe lamp (λ > 420 nm)	Degradation of CIP	DE = 92.6% (120 min)	**Deng et al. (2018)**
g-C_3_N_4_	LaFeO_3_	Ag	300 W Xe lamp (λ > 420 nm)	Degradation of MB	DE = 98.97% (90 min) DE = 92.93% (120 min)	**Zhang et al. (2021a)**
				Degradation of TC		
g-C_3_N_4_	AgVO_3_	Ag	300 W Xe lamp (λ > 400 nm)	Degradation of RhB	DE = 100% (12 min)	**Liu et al. (2019)**
				*E. coli* inactivation	3.05 log (100 min)	
g-C_3_N_4_	AgCl	Ag	300 W Xe lamp (λ > 420 nm)	Degradation of Rh B	DE = 100% (60 min)	**Bao and Chen (2016)**
				Degradation of MO	DE = 99% (90 min)	
g-C_3_N_4_	Ag_2_CrO_4_	Ag	500 W Xe lamp	Degradation of MO	DE = 78% (30 min)	**Yu et al. (2021)**
g-C_3_N_4_	TiO_2_	Ag	500 W Xe lamp	Reduction of U (VI)	99% (30 min)	**Liu et al. (2022)**
g-C_3_N_4_	Zn0.5Cd0.5S	Au	300 W Xe lamp (λ > 420 nm)	Reduction of CO_2_ for CH_3_OH evolution	1.31 μmol h^−1^ g^−1^	**Madhusudan et al. (2021)**
g-C_3_N_4_	CdS	Au	300 W Xe lamp (λ > 455 nm)	H_2_ production reduction of CO_2_	277 μmol h^−1^ (4 h)	**Zheng et al. (2015b)**
			300 W Xe lamp (λ > 420 nm)		85%	
g-C_3_N_4_	ZnIn_2_S_4_	Au	300 W Xe lamp	Removal of NO	59.7%	**Zhang et al. (2020a)**
				CO production	242.3 μmol h^−1^ g^−1^	
g-C_3_N_4_	AgCl	Au	200 W Xe lamp (λ > 420 nm)	Degradation of Rh B	DE = 93.1% (25 min)	**Zhang et al. (2021b)**
g-C_3_N_4_	TiO_2_(P25)	Au	150 W Hg Lamp	H_2_ production	419 μmol h^−1^ g^−1^	**Jiménez-Calvo et al. (2020)**
g-C_3_N_4_	Cu_2_ZnSnS_4_	Pt	400 W Xe lamp (λ > 420 nm)	Reduction of CO_2_ for CO/CH_4_ evolution	17.351/7.961 μmol h^−1^ g^−1^	**Raza et al. (2020)**
g-C_3_N_4_	AgVO_3_	Pt	300 W Xe lamp (λ > 420 nm)	H_2_ production	10,444 μmol h^−1^ g^−1^	**Qureshi et al. (2023)**
g-C_3_N_4_	BiVO_4_	Pt	300 W Xe lamp (λ > 420 nm)	Degradation of MB	DE = 100% (70 min)	**Si et al. (2020)**
				Degradation of BPA	DE = 92.7% (130 min)	
				H_2_ production	72 μmol h^−1^ g^−1^	
g-C_3_N_4_	WO_3_	C	500 W Xe lamp (λ > 420 nm)	Degradation of TC	DE = 75% (60 min)	**Zhao et al. (2021)**

DE, degradation efficiency; E, efficiency; Rh B, Rhodamine B; TC, tetracycline; SMZ, sulfamethoxazole; NO, nitric oxides; CIP, ciprofloxacin; MB, methylene blue; MO, methyl orange; BPA, Bisphenol A; 2,4-DCP, 2,4-dichlorophenol; TC-HCl, Tetracycline Hydrochloride; CR, congo red; GO, graphene oxide; RGO, reduced graphene oxide; AFB_1_, Aflatoxins B_1_.

The publisher apologizes for this mistake. The original version of this article has been updated.

